# Incidence of Inborn Errors of Metabolism and Endocrine Disorders Among 40965 Newborn Infants at Riyadh Second Health Cluster of the Ministry of Health Saudi Arabia

**DOI:** 10.3390/ijns10040072

**Published:** 2024-10-16

**Authors:** Abdullah S. Alshehri, Abdul A. Peer-Zada, Abeer A. Algadhi, Abdulwahed Aldehaimi, Mohammed A. Saleh, Aziza M. Mushiba, Eissa A. Faqeih, Ali M. AlAsmari

**Affiliations:** 1 Section of Biochemical Genetics and Toxicology, Pathology and Clinical Laboratory Medicine Administration, King Fahad Medical City, Riyadh 11525, Saudi Arabia; azada@kfmc.med.sa (A.A.P.-Z.); aalkadi@kfmc.med.sa (A.A.A.); aaldehaimi@kfmc.med.sa (A.A.); 2 Section of Medical Genetics, Department of Pediatrics, King Fahad Medical City, Riyadh 11525, Saudi Arabia; msaleh@kfmc.med.sa (M.A.S.); amushiba@kfmc.med.sa (A.M.M.); efaqeih@kfmc.med.sa (E.A.F.)

**Keywords:** newborn screening, dried blood spots, inborn error of metabolism, tandem mass spectrometry

## Abstract

Inborn errors of metabolism (IEM) and endocrine disorders are common genetic conditions in the Saudi population with the incidence rate often underestimated. Newborn screening (NBS) using various disease panels provides the first line in the early detection and intervention among infants with a high risk of IEM. Here we aim to assess the incidence of screening disorders and provide an overview of the NBS program at the Ministry of Health Tertiary Care King Fahad Medical City. Dried blood spots (DBS) from 40,965 newborn infants collected on the second day after birth were analyzed for 20 disorders. The total number of positive screen (“repeat”) samples over 10 years was about 1% (*n* = 382/40,965). The true positive result rate was 15.3% (*n* = 46/301) with the recall rates of individual disorders ranging from 0.26% (95% CI, 0.17–0.69) to 2.6% (95% CI, 2.19–3.05). The false positive result rate was 84.7% (*n* = 255/301) with biotinidase activity found to be the most common cause of the second sample repeat. The overall incidence of the screened diseases was 1:891 (95% CI, 11.61–12.47). CH and CAH are the most prevalent among endocrine disorders with an incidence of 1:4097 (95% CI, 2.19–3.05), and PA and ASA among the IEM with an incidence of 1:10,241 (95% CI, 0.09–0.95). In summary, we provide updated data and our experience on the incidence of various IEM and endocrine disorders among the Saudi population, highlight the role of false positive results of biotinidase activity that can increase the recall rate and lead to overestimation of the incidence data, and recommend multicenter studies to achieve a successful national NBS program.

## 1. Introduction

Newborn screening (NBS) is a comprehensive system, consisting of screening, confirmatory testing, follow-up of abnormal results, and treatment/management assessments. The main goal of NBS is to identify seemingly healthy babies that, in fact, have inborn errors of metabolism (IEM) or one of the inherited diseases. Early detection of such disorders is important in preventing complications, such as neurological damage or developmental delay. Screening programs for IEM have already been applied for several years worldwide and have indeed significantly helped to reduce infant morbidity and mortality [1]. There are actually ~50 diseases that can now be screened and the list is growing over the years. However, there is a considerable variation in the incidence and the number of screened disorders among countries [2]. For example, IEM are reported to occur globally in 1:1500 or 50.9:100,000 live births [3,4] and in Saudi Arabia, the overall incidence of IEM reported thus far is 1:1043 and 1:1490 [5,6]. The national NBS program in the Kingdom of Saudi Arabia (KSA), which was initiated in 2005 to screen for IEM and endocrine disorders, screens for 20 analytes [7].

Historically, PKU was established as the universal IEM to be tested using DBS [8]. Subsequently, immunoassays for thyroid function were developed and integrated into the NBS panel [9,10]. The introduction of tandem mass spectrometry (MS/MS) in neonatal screening programs revolutionized NBS by detecting a large number of different analytes in a short period of time. The MS/MS is capable of quantifying marker metabolites of amino acidemias, organic acidurias, and fatty acid oxidation disorders, namely amino acid and acylcarnitine simultaneously [11,12]. The screen panels of NBS are known to be population-based with 29 disorders screened in the USA and NBS in the KSA includes 17 inherited metabolic diseases and endocrine disorders and has in fact been updated recently in 2024 to include 20 diseases (Arabic/English version https://www.moh.gov.sa/Ministry/About/Health%20Policies/National-NBS-Program.pdf, accessed on 1 April 2024). Although NBS is performed in many hospitals within the KSA, only a few centers have reported the data leading to the underestimation of the incidence of IEM among the Saudi population [5,6,13].

In the current study, we included 40,965 newborns whose DBS were screened for disorders of IEM at KFMC from 2012 to 2021. This study is one of the largest retrospective analyses of newborn screening amongst the neonate cohort from a single center within the Ministry of Health in the KSA.

## 2. Methodology

DBS: This retrospective study was carried out on all 40,965 infants who underwent NBS in the KFMC, Riyadh, Saudi Arabia, from 2012 to 2021. The samples were collected on the second day of life by certified nurses after completing the infant information on the card, including gestational age, sex, mother’s medical record number, and birth weight, and indicating if it was a repeat specimen. The information provided was checked with the identification band on the infant. All precautions were followed, such as wearing gloves throughout the procedure, cleaning the baby’s heel with an alcohol swab, and wiping it with sterile gauze to dry the area. Then, a sterile lancet was used to prick the heel, and the first blood drop was wiped away to obtain the second large hanging drop. One drop was applied to the center of the five circles of the filter paper to ensure it filled the whole circle. After collection, the filter paper cards were placed on a flat surface at a room temperature of 18–25 °C to dry before being sent to the Department of Biochemical Genetics laboratory. In addition, it was ensured that the cards did not touch each other during packaging and transportation. Thereafter, and before the analytical phase and after the cards’ arrival, specimen integrity was checked visually by a laboratory technologist for mismatched patient identifiers (name and medical record number), insufficient specimen quantity, scratched specimens, non-dry specimens, supersaturated specimens, presence of serum rings in the specimens, clotted or contaminated specimens and no blood spots. In the case of any non-compliance, a new specimen was requested promptly. Then one 3.2-mm disc was punched from the DBS sample into a 96-well plate. The tests were performed on a separate 96-well plate for each assay.

College of American Pathologists/Clinical Biochemical Genetics standards were applied throughout all laboratory procedures to ensure high-quality results. In addition, since 2012, the Department of Biochemical Genetics has participated in the CDC’s Newborn Screening Quality Assurance Program (NSQAP).

Tandem MS/MS: The amino acids, succinylacetone, and acylcarnitine were analyzed using an in-house method with derivatization on a tandem mass spectrometer (SCIEX API 2000 and SCIEX API 3200, Hartford, CT, USA). Briefly, a 3.2 mm blood spot was extracted with methanol containing a stable isotope internal standard and derivatized with butanol-HCl [11,14]. Samples were measured by electrospray ionization–mass spectrometry (ESI-MS/MS) in positive ionization mode. The metabolites were detected using either the 102 neutral loss, 85 pre-cursor ion, or multiple reaction monitoring acquisition mode (MRM).

Fluorometric assays: Neonatal Biotinidase kit (part number 3018-0010), DELFIA neonatal 17-alpha-hydroxyprogesterone kit (part number A024-110), DELFIA neonatal hTSH kit(part number A032-310) and Neonatal GALT kit (part number NG-1100) were analyzed on a VICTOR2 D, Revvity (Turku, Finland) instrument.

Certified clinical scientists reported and interpreted the results based on an established in-house cut-off where more than one thousand specimens were analyzed for each analyte and concluded by eliminating false negatives while adjusting the false positive rate. In addition, they were compared with published references (Table 1). Furthermore, the cut-off was continually reviewed and readjusted if necessary. Based on the cut-off, the sample was assigned normal, abnormal, or questionable status. For presumptive false positives and abnormal results, the standard protocol requires that once an infant is identified with a suspicion of one of the disorders, the staff must notify the physician to collect a second sample for confirmation. The confirmatory analysis was carried out in either urine to detect the organic acid profile using gas chromatography–mass spectrometry (GC-MS) or in plasma for an amino-acid profile using an ultra-performance liquid chromatography technique.

Newborn screening using MS/MS cannot distinguish between some diseases due to the utilization of the same markers. For example, an elevation in C5OH concentration is an indicator for a wide range of diseases. Therefore, in this study, urine organic acid is useful to distinguish between HMG and 3MCC. In the same manner, the elevation of C3 and C3/C2 ratios may indicate MMA or PA. The urine organic acid profile can be used to differentiate.

Recall rate and 95% CI was calculated using the following formulae:

Recall rate (%) = (number of confirmed cases/total number of positive screens) × 100 95% CI = Mean ± (1.96 × standard deviation/√*n*)

## 3. Results

**Figure 1:**
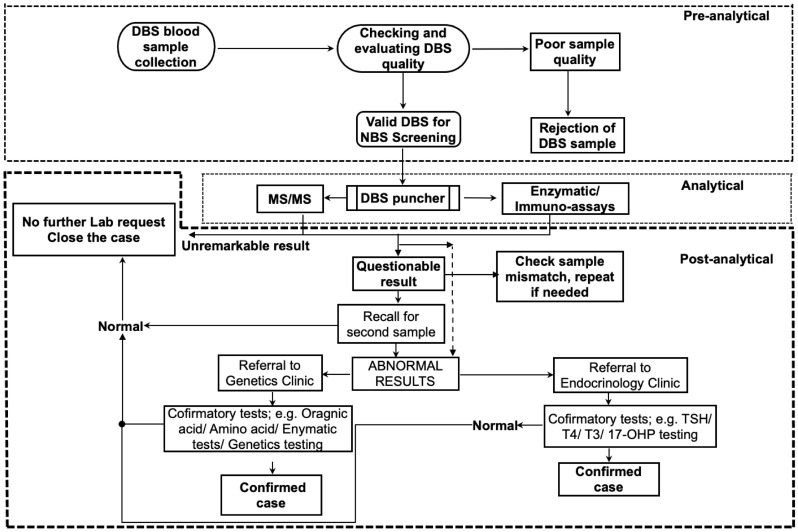
The algorithm used at KFMC shows the NBS workflow. A DBS blood sample is collected followed by a pre-analytical quality check (QC) to assess the validity of the specimen for NBS. DBS is extracted with a single punch into a single well of a 96-well microplate. Tandem mass spectrometry (MS/MS) for the amino acids, succinylacetone, and acylcarnitine is performed using a derivatized-based method on an API 2000 and API 3000 tandem MS. The fluorometric assay: biotinidase activity, 17-hydroxyprogesterone, thyroid stimulating hormone (TSH), and galactose-1-phosphateuridyl transferase (GALT) are on a VICTOR2 D, Revvity (Turku, Finland). The QC is used to validate the entire process of testing (details in the text).

At KFMC, a panel of 20 metabolic disorders was used for NBS (Table 2) and a well-established screening algorithm (Figure 1) was used to report the cases. We had a total of 40,965 newborn infant live births between 2012 to 2021 at KFMC. The total number of positive screen (repeat) samples over 10 years was 1% (*n* = 382/40965). When a presumptive positive screening result occurred, a second DBS was requested to provide confirmation. The second sample for confirmation was received from about 78.6% (*n* = 301/382) and not received from about 22% (*n* = 81/382). The number of confirmed abnormal test results was *n* = 46 samples, thereby giving a total recall rate of 12.04% (95% CI, 11.6–12.47). The number of confirmed results for each disorder with recall rates are shown (Table 3) and ranged from 0.26% (95% CI, 0.17–0.69) to 2.6% (95% CI, 2.19–3.05). The true positive and false positive percentage was observed to be 15.3% (*n* = 46/301) and 84.7% (*n* = 255/301), respectively. The majority of false positive results (*n* = 117/382) were observed to be due to biotinidase activity and only 2 were confirmed as abnormal results giving about a 30% false positivity rate (*n* = 115/382). With the initial cut-off at 44 U, the total number of questionable cases was 297 of which 94 were due to biotinidase activity giving about a 31% false positivity rate (*n* = 92/297). With the modified cut-off at 37 U, the total number of questionable cases was 85 of which 23 were due to biotinidase activity giving about a 27% false positivity rate (*n* = 23/87), resulting in about a 4% reduction in the false positive results.

There were 46 abnormal cases identified, giving an overall incidence of 1:891 (95% CI, 11.61–12.47) for screenable diseases (Table 3). The incidence of IEM in this study was 1:1576 (95% CI, 6.38–7.24). PA and ASA were common diseases with an incidence of 1:10241 (95% CI, 0.62–1.48). MCAD and GALT showed a similar incidence of 1:13655 (95% CI, 0.35–1.22). On the other hand, the endocrine disorders, CAH and CH, were the most frequent diseases among the population with a total incidence of 1: 2048 (95% CI, 4.80–5.67), and an individual incidence of each at 1:4097 (95% CI, 2.19–3.05).

It may be noted here that our laboratory at KFMC is CAP accredited and requires CAP proficiency testing (PT) or other external quality assurance program. We participate in the CDC’s Newborn Screening Quality Assurance Program (NSQAP) to maintain technical staff proficiency and competence on a periodic basis.

## 4. Discussion

We report on our 10 years of experience with NBS involving 40,965 neonates from the Riyadh Second Health Cluster of the Ministry of Health, Saudi Arabia. The Second Health Cluster consists of KFMC, and other hospitals in addition to primary care centers. We provide updated data on the incidence of various IEM and endocrine disorders among the Saudi population and highlight the role of false positive results of biotinidase activity that can dramatically increase the recall rate and lead to an overestimation of the incidence data. In Saudi Arabia, the NBS program was officially inaugurated by the MOH in August 2005 through a Royal decree to screen newborns across the Kingdom for early detection of hereditary diseases thought to cause serious health complications, thereby, posing a huge social and mental burden on the child’s parents. The program covers 183 hospitals across the country for screening all newborns in their first 24–72 h and helps detect abnormalities early on so as to provide the necessary clinical management as soon as possible and reduce the resulting morbidity, mortality, and disability [15]. Based on the disease prevalence data at of time of its inception, the NBS program in Saudi Arabia initially included 16 and later 17 diseases. [6] and was recently upgraded in 2024 to include 20 diseases. The assessment and effectiveness of the national NBS program have been previously described and more focus on the NBS public awareness program has been proposed in Vision 2030 [16,17]. There is generally a high acceptance and implementation of NBS in the Saudi population [18].

We analyzed 40,965 newborns with a presumptive positive screening result in about 1% (*n* = 382/40965). The second sample for confirmation was not received from about 22% (*n*= 81/382). Various factors contributed to the inability to secure a second sample, such as communication with the parents and some of the families preferring to follow up in other centers that were closer to where they lived. The recall rate for various disorders ranged from 0.26% (95% CI, 0.17–0.69) to 2.6% (95% CI, 2.19–3.05), which is consistent with other studies [19,20]. It is evident from multiple NBS studies that specific screening disorders, demographic locations, testing methodologies, and follow-up protocols can influence recall rates. The development of advanced technologies and unifying protocols across the globe will help reduce recall rates. Since follow-up is an important component of the NBS program, our laboratory informs the physician as soon as the result is confirmed to be positive through the NBS coordinator, who then will call the family to come for the next day’s metabolic or endocrine clinic, wherein the genetic pediatrician evaluates the infant and starts the management according to the standard protocol. Dieticians and genetic counselors are also involved in the same setting. Molecular confirmation and follow-up are requested for prevention and treatment strategy by the clinical side. We show an overall incidence of 1:891 (95% CI, 11.61–12.47), which is slightly different when compared with the other studies from different centers in Saudi Arabia. The increased incidence in our study could be attributed to the family history of a metabolic disorder, with the couples being advised to have their next babies delivered in the same center. The first study involving 165,530 infants that were born at Saudi Aramco Healthcare reported the estimated frequency of IEM at 1:667, which is higher than in our study [13]. It may be noted that a strict comparison between the two cannot be made since our NBS panel includes additional disorders not included in the Aramco study, such as glycogen storage, lysosome storage, mitochondrial, and other IEM disorders. A broad retrospective study by Alfadhel M et al., among 775,000 newborns, showed the overall incidence of the screened disorders to be 1:1043 [5]. Another cohort by Mohamed S et al., involving 56,632 infants from Prince Sultan Military Medical City (PSMMC), Riyadh, showed the incidence to be 1:1490, which is similar to that of the previous study [6].

The incidence of screened IEM was 1:1576 (95% CI, 6.38–7.24), the PA and ASA being the most frequently detected disorders with an incidence of 1:10,241 (95% CI, 0.62–1.48), which is in fact, one of the highest incidences reported among the Saudi population so far. Interestingly, GALT incidence was 1:13,655 (95% CI, 0.35–1.22), which is comparable with the incidence of 1:14,245 reported by Alfadhel et al., GALT incidence was reported to be much lower at 1:56,632 by Mohamed et al. [6]. Our study, unlike other studies, has shown a high incidence of MCADD at 1:13,655 (95% CI, 0.35–1.22) and HMG at 1: 20,483 (95% CI, 0.09–0.95), which is the highest incidence to have been recorded for MCADD in the KSA. There is either a low incidence or no incidence at all of some diseases among the studied population in our cohort. The incidence of endocrine disorders (CAH and CH) was 1:2048 (95% CI, 4.80–5.67) in our study with CH at 1:4097 (95% CI, 2.19–3.05), which is close to the findings by Mohamed et al., and Al-Jurayyan et al., who showed that the incidence of CH in Saudi Arabia to be 1:3775 and 1:3417, respectively [6,21]. They, however, did not report the incidence of CAH.

Abnormal biotinidase activity was detected in a large number of false positives and partially deficient cases. There are several factors that are associated with such a false-positive result. First, biotinidase enzyme activity may have been affected by the health status of the neonate. Jaundice, for example, has been reported to be capable of affecting the enzyme activity [22]. Second, a lack of cut-off values may contribute to increased false positivity. Khan et al. recently reported clinical disease ranges for most of the Saudi NBS analytes and several of the ratios used to improve screening specificity and sensitivity [23]. Reducing the cut-off values could minimize reporting false positive results thereby impacting unwanted referrals and concerns, parental stress, and anxiety. Based on our population reference range for biotinidase enzyme activity screening, we updated the cut-off value to 37 U instead of 44 U. The cut-off was updated in the year 2019, after 7 years and 6 months, which resulted in about a 4% reduction in false positive cases for biotinidase deficiency. Third, rescoring the second sample when the first sample was presumptive positive for any screened disease is also described by Alfadhel et al. [5]. The rescoring of the second sample was only 51% compared with 78.8% in our study. In our study, the samples also arrived at the newborn screening laboratory within two working days, which considerably reduced the risk of sample deterioration due to prolonged dispatch. A robust communication between the laboratory and the clinical team would be essential to enable second samples to be received without delay. Regarding false-negative NBS results, several studies have been described that attribute such results to the factors related to the time of sample collection, blood transfusion, dilute specimen, storage condition delay in sample processing, instrument sensitivity, and cut-off value [24,25]. We did not encounter any false negative results. With the advancements in the next generation sequencing technologies, shortcomings in the NBS program with regard to false positive/negative rates could be easily overcome [26].

Our experience with the NBS program has shown reduced infant mortality and morbidity through early interventions by the treating physicians in detected positive cases. A major difficulty in implementing the program has been in communication with the families who come from remote areas, a lack of response from parents, and compliance issues for long-term follow-up leading to the exclusion of infants for screening. Country-wide educational and awareness campaign, social media coverage, and visibility of the program and its usefulness for the community needs to be promoted in order to successfully implement NBS. Digitalization of health care records with a unified medical record number in the Kingdom is improving, which will also impact the performance of the NBS program. Tracking families for long-term follow-up to see the successful implementation of the program needs to be improved. Furthermore, having a national program to record the data from different centers would be extremely helpful, enabling clear evidence to be stored and explored and helping decision-makers to update the current NBS panel in Saudi Arabia. It is extremely useful to have national guidelines for NBS standards, including a well-defined procedure from the collection of DBS to the interpretation of the results. The effectiveness of NBS needs a key performance indicator (KPI) for monitoring the pre-analytical, analytical, and post-analytical processes.

In summary, NBS programs are expanding and becoming an important public health prevention strategy globally. The success in reducing morbidity and mortality through NBS will ultimately depend upon the focus on the outcome of NBS from different NBS centers, multicenter communication, and coordination to establish a basic database and overcome the overestimation or underestimation of the screened disorders amongst the Saudi population.

## Figures and Tables

**Table 1:** Cut-off values for each analyte used at the KFMC NBS program.

Amino Acids and Acylcarnitine					
Analyte	Cut-off Value	Initial Cut-off	Analyte	Cut-off Value	Initial Cut-off
C0 (μmol/L)	>5.83	>8 *^1^	Citrulline (μmol/L)	<37	<55 *^1^
C2 (μmol/L)	>7.5	-	Glutamine (μmol/L)	<707	-
C3 (μmol/L)	<6.75	<5.65 *^1^	Glycine (μmol/L)	<739	-
C3-DC (μmol/L)	<0.32	<0.25 *^1^	Methionine (μmol/L)	<61	<75 *^1^
C4 (μmol/L)	<0.85	<1.3 *^1^	Ornithine (μmol/L)	<284	-
C4-DC (μmol/L)	<0.64	-	Phenylalanine (μmol/L)	<130	<150 *^1^
C4-OH (μmol/L)	<0.48	<0.65 *^1^	Proline (μmol/L)	<263	-
C5 (μmol/L)	<0.86	<0.7 *^1^	Tyrosine (μmol/L)	<200	<350 *^1^
C5:1 (μmol/L)	<0.22	<0.25 *^1^	Valine (μmol/L)	<240	<300 *^1^
C5-DC (μmol/L)	<0.35	<0.35 *^1^	Xle (μmol/L)	<301	<290 *^1^
C5-OH (μmol/L)	<0.44	<0.8 *^1^	Arginine (μmol/L)	<44	<70 *^1^
C6 (μmol/L)	<0.26	<0.4 *^1^	Argininosuccinic (μmol/L)	<0.5	-
C8 (μmol/L)	<0.27	<0.45 *^1^	Aspartate (μmol/L)	<136	-
C10 (μmol/L)	<0.21	<0.45 *^1^	Alanine (μmol/L)	<689	-
C10:1 (μmol/L)	<0.18	<0.3 *^1^	Phe/Try Ratio	<1.8	-
C12 (μmol/L)	<0.39	-	Met/Phe Ratio	<2	-
C14 (μmol/L)	<0.84	<0.75 *^1^	Orn/Cit Ratio	<18	-
C14:1 (μmol/L)	<0.65	<0.6 *^1^	Cit/Arg Ratio	<6.7	-
C16 (μmol/L)	<7.19	<7.5 *^1^	Tyr/Phe Ratio	<3.9	-
C16-OH (μmol/L)	<0.15	<0.13 *^1^	Xle/Phe Ratio	<5	-
C18 (μmol/L)	<2.3	<2.3 *^1^	Arg/Orn Ratio	<0.42	-
C18:1 (μmol/L)	<3.16	<3.5 *^1^	Biotinidase activity (U)	>37	>48 *^2^
C18-OH (μmol/L)	<0.1	<0.1 *^1^	GALT (U/g Hp)	>4.7	>3.5 *^2^
C3/C1 Ratio	<0.27	-	TSH (μU/mL)	<20	<30 *^1^
C3/C2 Ratio	<0.24	-	17-OH-Progesterone (nmol/L)	<42	<35 *^1^
C8/C2 Ratio	<0.02	-	Succinylacetone (μmol/L)	<1.5	<2 *^1^
C8/C10 Ratio	<4	-			

C0, Free carnitine; C2, Acetylcarnitine; C3, Propionylcarnitine; C3DC, malonylcarnitine; C4, Butyryl/isobutyrylcarnitine; C4DC, Methylmalonylcarnitine; C4OH, 3-hydroxybutyrylcarnitine; C5, isovaleryl/2-methyl-butyrylcarnitine; C5DC, Glutarylcarnitine; C5:1, Tiglyly/3-methylcrotonylcarnitine; C5OH, 3-hydroxyisovalerylcarnitine; C6, Hexanoylcarnitine; C8, Octanoylcarnitine; C10, Decanoylcarnitine; C10:1, decenoylcarnitine; C12, Dodecanoylcarnitine; C14, Myristoylcarnitine; C14:1, Tetradecenoylcarnitine; C16, palmitoylcarnitine; C16OH, 3-hydroxyhexadecanoylcarnitine; C18, Stearoylcarnitine; C18:1, Oleylcarnitine; C18OH, 3-hydroxyoctadecanoylcarnitine; Met, Methionine; Orn, Ornithine; Xle, leucine/isoleucine; Arg, Arginine; Phe, Phenylalanine; Tyr, Tyrosine; GALT, Galactose-1-phosphate uridyltransferase; TSH, Thyroid Stimulation Hormone. *^1^ NSQAP—CDC Cut-off; *^2^ Manufacture recommended cut-off.

**Table 2:** KFMC in-house NBS panel including 20 disorders.

	Disorder	Abbreviation	Marker
	Endocrine Disorders		
1.	Congenital Hypothyroidism	CH	Thyroid Stimulation Hormone
2.	Congenital Adrenal Hyperplasia	CAH	17-OH-Progesterone
	Amino Acids Disorders		
3.	phenylketonuria	PKU	Phenylalanine, Phe/Tyr
4.	Maple Syrup Urine Disease	MSUD	Valine, Xle, Xle/Phe
5.	Tyrosinemia Type I	TYR I	Tyrosine, Succinylacetone
6.	Homocystinuria	HCY	Methionine, Homocystine
	Organic Acidurias		
7.	Propionic Acidemia	PA	C3, C3/C2
8.	Methylmalonic Acidemia	MMA	C3, C3/C2
9.	3-Methylcrotonyl-CoA Carboxylase Deficiency	3MCC	C5OH
10.	3-Hydroxy-3-Methylglutaric Acidemia	HMG	C5OH
11.	Beta-Ketothiolase Deficiency	BKD	C5:1, C5OH
12.	Isovaleric Acidemia	IVA	C5
13.	Glutaric Acidemia Type I	GA I	C5DC
	Urea Cycle Disorders		
14.	Argininosuccinic Acidemia	ASA	Argininosuccinic acid, Citrulline
15.	Citrullinemia	CIT	Citrulline
	Fatty Acid Oxidation Defects		
16.	Medium Chain Acyl-CoA Dehydrogenase Deficiency	MCADD	C6, C8, C10, C10:1, C8/C2, C8/C10
17.	Very Long Chain 3-Hydroxyacyl-CoA Dehydrogenase Deficiency	VLCAD	C14, C14:1
18.	Primary Carnitine Deficiency	CUD	C0
	Disorders of Carbohydrate Metabolism		
19.	Galactosemia	GALT	Galactosemia-1-phosphate uridyltransferase
20.	Biotinidase Deficiency	BTD	Biotinidase

C0, Free carnitine; C2, Acetylcarnitine; C3, Propionylcarnitine; C3DC, malonylcarnitine; C4, Butyryl/isobutyrylcarnitine; C4DC, Methylmalonylcarnitine; C5, isovaleryl/2-methyl-butyrylcarnitine; C5DC, Glutarylcarnitine; C5:1, Tiglyly/3-methylcrotonylcarnitine; C5OH, 3-hydroxyisovalerylcarnitine; C6, Hexanoylcarnitine; C8, Octanoylcarnitine; C10, Decanoylcarnitine; C10:1, decenoylcarnitine; C12, Dodecanoylcarnitine; C14, Myristoylcarnitine; C14:1, Tetradecenoylcarnitine; C16, palmitoylcarnitine; C16:1, hexadecenoylcarnitine; C18, Stearoylcarnitine; C18:1, Oleylcarnitine;

**Table 3:** The number and incidence of screened disorders.

	List of Diseases	Number of Confirmed Cases	Incidence	Recall Rate (%)	95% CI
Endocrine Disorders					
1.	CAH	10	1:4097	2.6	2.19–3.05
2.	CH	10	1:4097	2.6	2.19–3.05
Total Positives (Overall incidence-Endo)		20	1:2048	5.24	4.80–5.67
Inborn Errors of Metabolism					
3.	PKU	1	1:40,965	0.26	0.17–0.69
4.	MSUD	2	1:20,483	0.52	0.09–0.95
5.	TYR1	-	-		
6.	HCY	-	-		
7.	PA	4	1:10,241	1.05	0.62–1.48
8.	MMA	1	1:40,965	0.26	0.17–0.69
9.	3MCC	-	-		
10.	BTD	2	1:20,483	0.52	0.09–0.95
11.	GA1	1	1:40,965	0.26	0.17–0.69
12.	IVA	1	1:40,965	0.26	0.17–0.69
13.	HMG	2	1:20,483	0.52	0.09–0.95
14.	BKD	-	-		
15.	ASA	4	1: 10,241	1.05	0.62–1.48
16.	CIT	2	1:20,483	0.52	0.09–0.95
17.	MCADD	3	1:13,655	0.79	0.35–1.22
18	VLCADD	-	-		
19.	CUD	-	-		
20.	GALT Deficiency	3	1:13,655	0.79	0.35–1.22
Total Positives (Overall incidence-IEM)		26	1:1576	6.81	6.38–7.24
Overall incidence (cumulative Endo-IEM)		46	1:891	12.04	11.61–12.47

## Data Availability

Data are contained within the article.

## Abbreviations

IEM	inborn errors of metabolism
NBS	newborn screening
DBS	dried blood spot
PA	propionic academia
MMA	methylmalonic acidemia
PKU	phenylketonuria
CH	congenital hypothyroidism
CAH	congenital adrenal hyperplasia
GALT	galactosemia
BTD	biotinidase deficiency
MCADD	Medium-chain acyl-CoA dehydrogenase deficiency
ASA	argininosuccinic acidemia
KSA	Kingdom of Saudi Arabia
CDC	Center for Disease Control
NSQAP	Newborn Screening Quality Assurance Program (CDC-based)
JCI	Joint Commission International
CAP	College of American Pathologists
MOH	Ministry of Health
KFMC	King Fahad Medical City
MS/MS	mass spectrometry (tandem)
KPI	key performance indicators
QC	quality control
NGS	next generation sequencing
